# Embodied echoes: navigating the familiarity in continued intention to play VR game

**DOI:** 10.3389/fnhum.2024.1495845

**Published:** 2024-11-20

**Authors:** Ling Yang, Daibo Xiao

**Affiliations:** ^1^Schools of Economics and Trade, Guangzhou Xinhua University, Guangdong, China; ^2^Faculty of Humanities and Social Sciences, City University of Macau, Macao, Macao SAR, China

**Keywords:** VR game, sense of familiarity, sense of embodiment, continued intention to play, perceived cost

## Abstract

**Introduction:**

Virtual reality (VR) games, propelled by advancements in VR and artificial intelligence technologies, offer a level of realism and interactivity that traditional games cannot match. However, despite their immersive potential, VR games have not yet reached the widespread popularity of their conventional counterparts. While VR can craft the illusion of a parallel reality, users often remain cognizant of the delineation between the virtual and the real.

**Methods:**

In this paper, we employ a blend of qualitative and quantitative research methods to explore the impact of familiarity with virtual environments and interactive elements on users sense of embodiment, flow experience, and their intention to continue playing VR games. Additionally, we examine the moderating influence of perceived cost within this framework.

**Results and discussions:**

Our analysis of 307 collected responses, facilitated by PLS-SEM, reveals that familiarity with interactivity is positively associated with both sense of embodiment and flow experience, whereas familiarity with the virtual scene primarily influences sense of embodiment. Interestingly, perceived cost exerts a positive moderating effect on the relationship between flow experience and the intention to persist with VR gaming, while it negatively moderates the impact of sense of embodiment on this intention. This study offers theoretical insights that can guide future research in the domain of VR gaming, as well as practical takeaways for companies in the VR game industry, shedding light on how to enhance user engagement and sustain long-term interest in VR gaming experiences.

## 1 Introduction

The Metaverse is capturing the attention of both scholars and businesses. It's a novel concept that allows people to create avatars and perform actions in a virtual world that are not possible in reality. With the advancements in VR and AI, users are entering the “Metaverse” era, which provides more immersive experiences. Users are transforming into active participants with an increased sense of presence, especially in gaming where they can explore and interact within virtual environments, leading to deep immersion. However, VR games have not yet seen the widespread adoption that traditional games have, often due to their inability to perfectly replicate real-life experiences. Users may struggle to perform actions that feel natural in the real world, which can disrupt the immersive experience. Therefore, improving the user experience in VR gaming, particularly the sense of fit between the virtual and real worlds, is an important area of study for enhancing the intention to continue playing VR games.

Previous studies have utilized the technology acceptance model to identify factors influencing VR adoption, including the technical aspects of VR and the demographic traits of users (Kosa et al., 2023). They have also concentrated on the technical and emotional aspects of VR, such as immersion, presence, empathy, and embodiment (Shin, 2022). Recently, a few studies have intriguingly found that a sense of familiarity (SOF), which involves implicit knowledge, can boost presence by leveraging users' past memories (Cerda et al., 2021). However, these studies fail to further categorize the SOF, explore its impact on users' ongoing intention to engage with VR games, or elucidate the underlying mechanisms. Additionally, several studies have shown the influence of perceived cost, including monetary and energetic investments, on VR usage behavior (Wang et al., 2018; Ye et al., 2020). Yet, with the falling prices of VR equipment and shifting user purchasing preferences, the role of perceived cost in affecting user behavioral intentions in VR gaming is not further illuminated. Therefore, our objective is to bridge this gap by investigating the factors that foster users' continued intention to play VR games. The specific questions we aim to address are as follows:

1. What contents are included in SOF within VR games?
2. How does SOF affect users' experience within VR games?
3. How do users' experience affects the continued intention to play VR games? And what is the role of perceived cost in this process?

For this, this study established a theoretical mechanisms and introduced sense of embodiment (SOE), which is the feeling of being present in one's avatar, is affected by how well the real and virtual worlds match up (Kilteni et al., 2012), the flow experience, perceived cost into the theoretical mechanisms to explain users experience of playing VR game. Our research employs a mixed-methods approach, combining qualitative insights with quantitative data analysis using Partial Least Squares Structural Equation Modeling (PLS-SEM). Through this, we examine a total of 307 responses to understand how different aspects of SOF affect users' SOE and flow experience, and how these, in turn, influence their intention to continue playing VR games.

In light of these considerations, this paper aims to delve deeper into the factors influencing the continued intention to play VR games and to offer potential recommendations for VR game designers. It primarily explores the concept of familiarity within VR games and the mechanisms linking familiarity to sustained play intentions, discusses the roles of SOE and flow experience, and their influence mechanisms. Additionally, this study reflects on the moderating role of perceived cost within this framework. The study is structured as follows: first, we review prior research to formulate hypotheses, then introduce our research methodology, including interviews and data analysis. Finally, we analyze our findings from interviews and questionnaires, offering insights and implications for professionals and researchers alike.

## 2 Literature review and hypothesis

### 2.1 The definition of SOF, SOE and flow experience

The SOF in psychological studies refers to implicit memory, a form of memory that operates without conscious awareness (Son et al., 2002). Cognitive psychologists Kaplan and Kaplan (1982, p. 92) define familiarity as the relationship between an individual and an entity with which they have had significant interaction. This interaction is substantial enough to influence various domains, including emotional, cognitive, and behavioral responses. In the context of VR gaming, individuals unfamiliar with VR or those viewing VR games as an alien environment may find adaptation challenging. Prior research indicates that people are more likely to adapt to and enhance their cognitive abilities in environments that are familiar, filled with known objects and sounds (Kaplan and Kaplan, 1982). Bozgeyikli and Bozgeyikli (2019) further suggest that the familiarity of interactions can mitigate the obstructiveness of technological tools. Thus, the sense of familiarity in VR games is cultivated not only through repeated exposure to elements like characters, objects, scenes, and sounds but also through natural interactions.

The SOE in VR environments involves a user's experience of body ownership, self-location, and agency (Tsakiris et al., 2006). In VR gaming, users perceive a virtual body as a replacement for their own, facilitated by sensory stimuli, and may even begin to imagine that this virtual body is an extension of their physical form. Scholars like Tsakiris et al. (2006) consider this phenomenon as body ownership. Botvinick and Cohen (1998) demonstrate that the ownership of an artificial body part can be induced through visual or tactile stimulation, leading to a synchronous perception of the physical hand. In essence, through such stimulation, participants start to perceive the artificial component as part of their body. This body ownership in virtual spaces can also be described as a self-mapped image that can independently perform body distribution and experience acquisition. Consequently, this self-mapped image is perceived as one's own physical body (Tsakiris et al., 2006). As a unique source of physical body perception, the virtual body exists within the spiritual life of a physical body (Tsakiris, 2010). In VR games, users manipulate game characters, and the real-time responses of these characters can lead users to feel a sense of ownership over the virtual limbs. The sensory stimulation of the virtual character seems to occur concurrently on the user's body, sharing the same perceptual source as the virtual character. As users perceive the virtual body as part of their own physical body, they can understand and control its movements. Lopez et al. (2008) suggest that the physical body can experience its location in a virtual space through the perception of the virtual body. While some scholars argue that this is similar to presence (Heeter, 1992), they are conceptually distinct; presence implies that individuals feel they are within the virtual world through their body sense, whereas self-location emphasizes disruptive body perception, requiring the experience of the virtual scene with the aid of a virtual body (Braun et al., 2018). In VR gaming, users often feel as if they inhabit a specific avatar from a first-person perspective, with a sense of bodily alignment and movement synchronization. Body ownership and self-location enable individuals to control the virtual body to perform desired actions. David et al. (2008) and Tsakiris et al. (2006) describe this as agency—the sense of controlling one's own body movements and interacting with the external environment through these controls. In VR gaming, agency means that users can manipulate the avatar's body as if it were their own, completing the game's tasks. To achieve a perfect embodiment with the avatar and a heightened level of embodied experience, VR game users should possess a strong sense of body ownership, agency, and self-location.

Flow theory, established by Csikszentmihalyi (2000), defines the flow experience as an optimal state of consciousness where individuals feel completely absorbed in an activity. Dawson (2017) posits that in VR games, flow represents the pinnacle of immersive experiences, with users' attitudes toward VR games potentially shifting from neutral to positive over time. If a game captivates users, they may gradually become immersed as the game progresses, losing track of time and ultimately reaching a state of flow. Current research often categorizes flow experience dimensions based on Csikszentmihalyi's (2000) concepts, including clear goals, immediate feedback, balance between skills and challenges, concentration, a sense of control, and a loss of self-awareness and time distortion. These dimensions are interconnected; for instance, focused attention can lead to a distorted perception of time.

Some scholars, like Skadberg and Kimmel (2004), define the flow experience as a blend of enjoyment and time distortion, while others, such as Senecal et al. (2022), categorize flow dimensions to include enjoyment, control, focus, and challenge. Shin (2017, 2018) describes the flow experience in AR or VR as a mental state where individuals forget the passage of time and their physical surroundings, becoming fully invested, enjoying, and losing themselves in the activity. Kim and Ko (2019) define the flow experience in VR live sports as a mental state encompassing enjoyment, cognitive absorption, and time distortion. Ahmad and Abdulkarim (2019) identify flow in gaming as the user's sense of immersion and presence within a virtual environment.

VR games offer realistic, 360-degree perspectives, fostering structural associations with real scenes and physical interaction experiences, including sensory experiences, spatial practices, identity replacement, emotional subjectivity, and false memories, which contribute to immersion (Kui, 2018). In the VR game world, through bodily interaction with the game system, users can easily become engrossed in the game scene, forgetting the passage of time and experiencing relaxation and joy, further empathizing with the emotions of the avatar characters. Under such conditions, users can escape real-world troubles and immerse themselves in the happiness that the game provides (He, 2021).

Thus, flow theory provides a theoretical foundation for understanding the dimensions of flow experience in VR games, which in this study include immersion, perceived control, and enjoyment.

### 2.2 The influence of SOF on SOE and flow experience

According to Kaplan and Kaplan (1982), the SOF elicits cognitive and emotional responses. Cognitively, being in a familiar environment stimulates embodied remembering, a process where the body's familiarity triggers sensory-based recollection (Sutton and Williamson, 2014). This SOF also accelerates metaphor comprehension, thereby enhancing embodiment (Jamrozik et al., 2016). In the realm of VR, Cai et al. (2018) discovered that familiar settings can aid memory recall, a finding echoed by researchers such as Cerda et al. (2021) and Osborne and Jones (2022). They noted that familiar contexts and interactions in VR can enhance the sense of presence and embodied familiarity.

Although the sense of embodiment, defined as “the feeling of being embodied in one's avatar” (Kilteni et al., 2012), differs from the definition of presence, which is “the feeling of being in the virtual world” (Schuemie et al., 2001), these concepts are closely intertwined (Fribourg et al., 2018; Shin, 2018). Lin (2017) suggested that interactivity in VR games can enhance the plausibility of events, influencing users' willingness to believe in the occurrence of these events. In this sense, plausibility arises from prior experiences creating a SOF when events reoccur.

Qureshi (2018) experiment demonstrated that participants felt a stronger sense of body ownership when the rubber hand was in a more familiar position, highlighting the link between familiarity and body location. Lei and Mou (2021) found that users' self-location can be updated with visual cues that provide spatial memory of familiar spaces. Although no studies directly link the sense of familiarity with agency, Zhang et al. (2020) showed that a sense of familiarity, created by a rubber hand's resemblance to the real hand and natural interaction, can improve the sense of control, hinting at a potential relationship between familiarity and agency.

Thus, in the context of VR gaming, it is plausible to suggest that familiar scenes and interactivity help users recall past memories, enhancing their belief in their presence within the virtual world and even their embodiment in game avatars. Consequently, we propose the following hypotheses:

H1a: The SOF with the scene is positively associated with the SOE.
H1b: The SOF with the interactivity is positively associated with the SOE.

Conversely, the flow experience can be seen as an emotional response to the sense of familiarity. Although no studies have explicitly examined the relationship between familiarity and flow in VR gaming, Douglas and Hargadon (2000) found that familiar scenes and narratives can immerse audiences in films and elicit more enjoyable emotions, a finding that could be extrapolated to the VR context. Familiarization can quicken metaphor comprehension, reducing technological barriers in VR gaming (Jamrozik et al., 2016), thus making users feel more enjoyment and immersion. Activities that defy real-world physics, like flying, may not convey realism and can detract from immersion (Ch'Ng et al., 2020). In contrast, when users feel familiar with scenes and activities, they are more likely to feel excited and inclined to share their experiences (Ch'Ng et al., 2020). Hence, we hypothesize:

H2a: The SOF with the scene is positively associated with the flow experience.
H2b: The SOF with the interactivity is positively associated with the flow experience.

### 2.3 The influence of SOE on continued intention to play VR game

Drawing from embodied theory, human physical experiences and cognitions arise from the dynamic interplay between the body and its environment (Shapiro, 2014). Even when the body is no longer in its original setting, these experiences and cognitions become integrated into the psychological fabric of an individual's broader cultural activities, thereby influencing subsequent behavioral patterns (Csordas, 1990, p. 30). Consequently, through the lens of embodied theory, the SOE can be intricately linked to subsequent behaviors. Scholars have delved into consumer behavior from an embodied perspective, recognizing SOE as a distinct experience that transcends emotional experiences, directly shaping cognition or perspective through the input and transformation of bodily sensory information (Valtonen and Närvänen, 2016). Yanli (2021) discovered that consumers can assimilate tactile sensory information via virtual tactile cues in online product displays, aligning closely with the avatars depicted in images to elicit embodiment, which in turn affects their purchase intentions. This finding is translatable to the realm of VR gaming, where a user's alignment with their in-game avatar fosters a robust SOE, allowing them to momentarily forget reality and become immersed in the game, enhancing their desire to play. Shin (2018) observed that VR can evoke embodied narrative engagement, heightening both emotional and physical involvement. Similarly, in VR gaming, high embodiment allows users to feel a congruence between their physical movements and in-game avatars, potentially increasing their engagement with the game.

While direct studies on the relationship between SOE and consumer behavior are sparse, research on embodied cognition offers valuable insights. Woermann and Rokka (2015) applied embodied cognition theory to understand the temporal experiences, bodily routines, and skills of individuals participating in freeskiing and paintball, affirming that the body is a wellspring of emotional expression and cognition, including skills, cultural knowledge, and techniques. With the evolution of information technology, many enterprises leverage new media to stimulate consumers' senses, prompting them to experience “body ownership,” “self-location,” and “agency.” Initially, companies introduced virtual advertisements, allowing users to view product images and movements within realistic settings, instilling a sense of agency (Caspar et al., 2015). Even the sensory stimulation from consumer body reflection can catalyze spiritual transformation, fostering a sense of ownership. The virtual world's product usage experience, detached from physical interaction, is becoming increasingly significant in consumer behavior (Lysik and Lopacinski, 2019). Despite the absence of physical product interaction, consumers report heightened satisfaction, which can trigger their intention to purchase (Lysik and Lopacinski, 2019). In contrast to traditional websites, VR technologies enable consumers to engage in simulated real environments (Luangrath et al., 2022; Alcañiz et al., 2019). VR game interactions with characters involve personal physical movements, transcending the use of a keyboard and mouse for navigation or zooming, and instead, mirroring real-world gestures with the body or body parts (Kilteni et al., 2012). Tsakiris et al. (2006) suggest that physical movement and sensory stimuli in VR, along with interactions with the external environment, elicit the SOE. Undoubtedly, this form of embodiment in gaming significantly enhances the user experience. Leveau and Camus (2023) also identified a positive correlation between embodiment and participation in virtual reality sports activities. Flavián et al. (2019) posit that high embodiment technology under VR can boost the willingness to select tourist destinations. Elder and Krishna (2012), pioneers in studying physical engagement in marketing, concurred that physical presence in a virtual body can influence behavioral intent. These scholars have demonstrated that the SOE stimulated by VR game interactions can precipitate user behavioral intentions to engage in gameplay. In other words, the stronger the SOE users experience in VR games, the more pronounced their consumption willingness, potentially leading to a sustained intention to play. Therefore, in this paper, we propose the following hypothesis:

H3. The SOE positively influences the continued intention to play VR games.

### 2.4 The influence of flow experience on continued intention to play VR game

In this paper, the flow experience is posited as a pivotal factor influencing the sustained intention to engage with VR games. Studies by Su et al. (2016) and Zhou (2019) have demonstrated that flow experience significantly enhances loyalty to gameplay through the medium of human-computer interaction. Furthermore, Zhou (2019) discovered that the flow experience on social network platforms positively influences consumer intent. Within the VR domain, Ahmad and Abdulkarim (2019) observed a robust correlation between flow experience and the intention to utilize VR applications. Similarly, in the gaming sector, Hao (2018) determined that flow experience influences users' immediate and future engagement with games. Su et al. (2016) also illustrated that flow is a catalyst for user loyalty. Drawing upon these findings, we propose the following hypothesis to elucidate the potential link between flow experience and the ongoing intention to play:

H4. Flow experience positively correlates with the continued intention to play VR game.

### 2.5 The influence of SOE on flow experience

In the realm of VR, a heightened engagement of the body, mind, and spirit fosters a more profound SOE, thereby enhancing users' focus and altering their perception of time (Dawson, 2017). In VR gaming, bodily engagement implies a continuous and dynamic interaction between users and their avatars, as well as the VR environment. Argelaguet et al. (2016) conducted experiments that revealed participants experienced a stronger sense of agency and more precise sensory feedback with more realistic and delicate virtual hands, as opposed to slightly crude ones. This finding underscores the significance of precise interaction with avatars in VR games, which can elicit positive bodily engagement and the belief that users can control the VR game with ease.

Jiang (2022) discovered that avatar identity mediates the relationship between the physical resemblance of avatars and the flow experience, as defined by perceived control. In essence, when a user's body not only mirrors the appearance of the avatar but also executes identical actions, the smoother the perceived control, highlighting the interplay between SOE and flow experience. Tussyadiah et al. (2017) posited that embodiment in virtual tourism settings can amplify tourists' perceived pleasure, consequently increasing the appeal of destinations. This principle is equally applicable to VR games, where users can physically align with virtual characters and become immersed in gameplay, experiencing the joy of the game. Consequently, SOE can intensify the user's flow experience, characterized by immersion, control, and enjoyment.

Thus, we propose the following hypothesis:

H5: SOE positively affects the continued intention to play VR game.

### 2.6 The moderating role of perceived cost

Perceived cost encompasses the monetary and non-monetary expenditures incurred by consumers when encountering new products or services (Amberg et al., 2004), which includes not only financial outlays but also the energy and effort expended (Ye et al., 2020). Within the scope of this paper, perceived cost is defined as the financial investment and energy expenditure that users incur while continuing to engage with VR games.

Perceived cost is recognized as a pivotal factor that influences consumer behavior, including acceptance, adoption, and even the formation of purchasing intentions (Wang et al., 2018). There is a natural inclination for individuals to pursue actions that demand minimal effort and financial input (Luarn and Lin, 2005; Wu and Wang, 2005). In the VR gaming sector, the costs associated with the utilization of VR games encompass not only the expense of the VR headset but also the energy spent on adjusting the device and securing the appropriate signal. Research has established that perceived cost is a significant determinant in the decision to adopt Internet of Things (IoT) technologies (Hsu and Lin, 2016), to opt for mobile coupons (Liu et al., 2015), and to utilize GPS navigation applications (Wang et al., 2018). The connection between perceived cost and behavioral intention is often mediated by perceived value, suggesting that users who are acutely aware of costs also seek to derive commensurate value (Liu et al., 2015).

While the majority of researchers concur on the generally negative influence of perceived cost on behavior or perceived value (Hsu and Lin, 2016; Liu et al., 2015), there are exceptions. Wang et al. (2018) discovered a positive relationship between perceived cost and the intention to use GPS navigation apps, and Vishwakarma (2020) found no correlation between perceived cost and perceived value in VR travel contexts. These findings underscore the need to explore the moderating role of perceived cost on the impact of SOE and flow experience on the continued intention to play.

Given the prevailing research that confirms the negative effect of perceived cost on behavior or perceived value, we propose the following hypotheses with an alternative perspective:

H6a: Perceived cost positively moderates the influence of SOE on the continued intention to play VR games.
H6b: Perceived cost positively moderates the influence of flow experience on the continued intention to play VR games.

**Figure 1:**
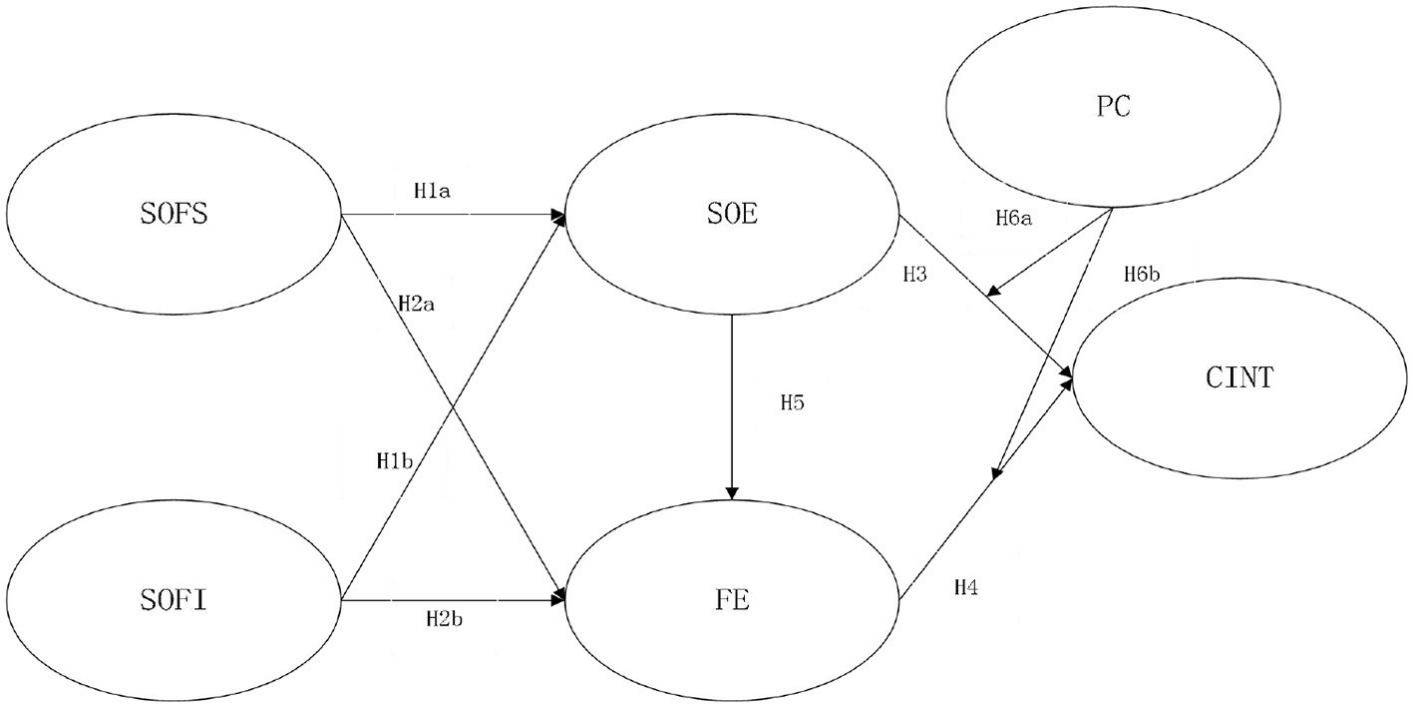
Structural model. SOE, sense of embodiment; CINT, continued intention to play; FE, flow experience; SOFI, sense of familiarity with the interactivity; PC, perceived cost; SOFS, sense of familiarity with the scene.

In brief, it is hypothesized that SOF influence SOE and flow experience so as to affect continuance intention of playing VR game and the overall model is provided in Figure 1.

## 3 Methodology

### 3.1 Procedure and scale developments

This section describes the procedures for data collection and the process of developing scales and associated questionnaires. To ensure content validity, it is crucial that the measurement items accurately reflect the essence of each construct and are aligned with the study's theme of VR gaming (Bohmstedt, 1970). Consequently, most of the measurement items in this study are adapted from previous research, then refined, and translated to suit the specific context of this study and its participants. However, due to the scarcity of existing literature and scales related to the SOF, this study initially uses a combination of semi-structured interviews and Cai et al. (2018)'s scale to inform the design before finalizing the scales and items.

We conducted semi-structured interviews with 25 undergraduate students (17 males and eight females) from universities in Guangdong province, China, using snowball sampling. Their interest in participating was due to their availability of free time and the opportunity to earn 20 RMB as a reward for the interview. This reward would cover half the cost of purchasing a favorite game in the Pico shop, a well-known Chinese VR game platform, or buying a cup of milk tea. The interviews incorporated open-ended questions that targeted the elements of SOF (e.g., “What aspects of the VR game do you find familiar?” “How similar is the VR game experience to that of PC gaming?”). The qualitative data collected through these interviews, documented using a recording device and transcribed into a written format, were then analyzed thematically using NVivo. This analysis aimed to uncover the underlying themes and perspectives of the participants. The coding process involved familiarizing ourselves with the text material, encoding and generating initial themes, and revising these themes in line with the corresponding literature. The detailed coding process is depicted in Table 1.

**Table 1:** Coding process.

**Theme**	**Subtheme**	**Coding**	**Original interview samples (partial)**
SOFS	Familiarity with the image	Exquisite picture	The details are very well done because I have been to the Mogao Grottoes, and it has captured many details that I didn't observe closely in Cave 220, which are incredibly realistic, as if they were in the real world
		Delicate animation effects	The visuals are incredibly realistic; when an empty magazine falls to the ground, it can roll to other places, just like how ordinary objects roll around everywhere when they drop
	Familiarity with the sound	Familiar background music	When I enter a very dark place, isn't there always some scary background music, just like when I watch a horror movie, it makes me feel afraid in an instant
		Familiar object sound	When firing at a glass bottle, I can hear the sound of the bottle shattering, just like the sound of us breaking glass on the ground
SOFI	Familiarity with responds from others	Familiar optional control	When I write with a calligraphy brush, it goes so smoothly and turns out so well, but it doesn't have the feeling from my childhood of worrying that a single stroke with the brush might make the lines too thick
		Familiar respond from enemy	After I shot the monster in the head, its reaction was very realistic
	Familiarity with reaction from avatar itself	Familiar Self-Motion	When I approach an obstacle, it prevents me from moving forward, just as I cannot walk through obstacles in a real environment
		Familiar avatar reaction	When I see a zombie, I instinctively retreat, and at this moment the game voices my inner monolog, such as “Damn it, don't come any closer”

After coding, the dimensions and the detailed items of SOF can be confirmed. Table 2 presents a summary of the constructs' definitions, item listings, and corresponding references. The survey items were reviewed by five experts, and the study team refined the items based on their feedback. The final items were formatted using a 5-point Likert scale, where “1” indicates “strongly disagree” and “5” signifies “strongly agree.”

**Table 2:** Definition and reference citation for valuables.

**Valuable**	**Definition**	**Items**	**Reference**
SOFS	The sense of familiarity is created by repeating and frequent exposing to environment such as roles, objects, scenes, or sounds etc in the VR game	I have seen things in the VR scene before and felt a sense of familiarity with it	Cai et al. (2018)
		The scene made me recall past memory	
		I have heard things in the VR scene before and felt a sense of familiarity with it	
SOFI	The sense of familiarity is created by natural interactivity	I felt a sense of familiarity with the action of avatar in the VR game	
		I felt a sense of familiarity with the reaction of avatar in the VR game	
		I felt a sense of familiarity with the controls	
		I felt a sense of familiarity with the reaction of targets (e.g. enemies) in the VR game	
SOE	The degree of being embodied in different body of avatar	You feel the virtual body parts you saw are your physical body parts	Galvan Debarba (2017)
		You think your virtual body reacts the same way your own body does	
		you can feel your body moving with the movement of the virtual body	
FE	A state of complete immersion and enjoyment in the present activity, often referred to as being “in the zone”	You forget about the passage of time during the game and feel that time is speeding up	Renard (2013)
		You know what you should do so as to play better	
		you think games are enjoyable	
CITP	The users' continued intention to play VR game	You're willing to keep playing VR game in the future	Hamari et al. (2017)
		You plan to play VR game instead of other games in the future	
		You are willing to pay to continue playing the game in the future if conditions permit	
PC	The users spend the cost and energy on the VR game	It is worth to pay for the cost of playing VR games in VR store	Ye et al. (2020)
		It is worth to pay for the cost of playing VR games at home	
		It is acceptable to consume energy to play VR game	

SOE, sense of embodiment; CINT, continued intention to play; FE, flow experience; SOFI, sense of familiarity with the interactivity; PC, perceived cost; SOFS, sense of familiarity with the scene.

**Figure 2:**
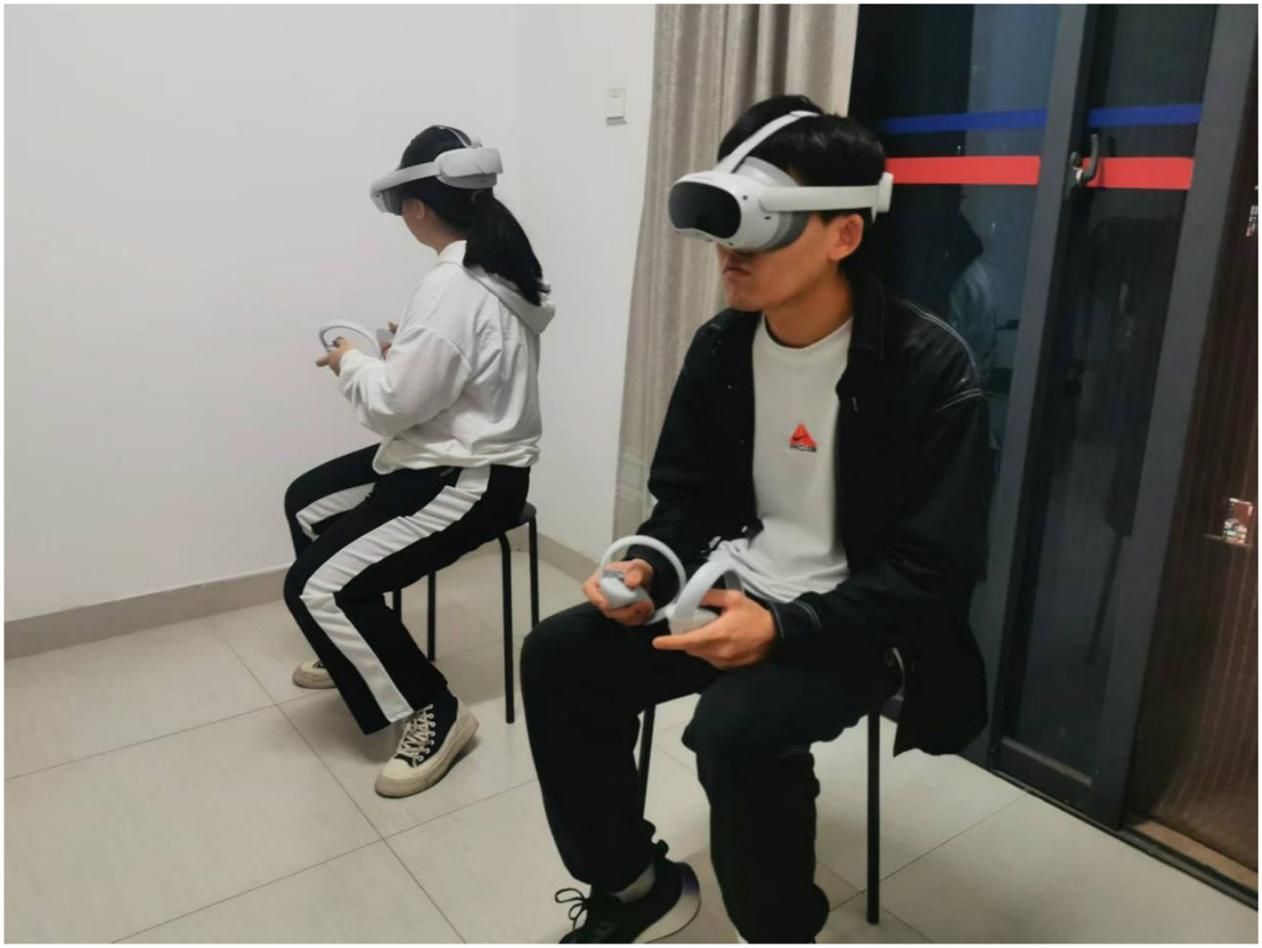
Gaming experience live.

**Figure 3:**
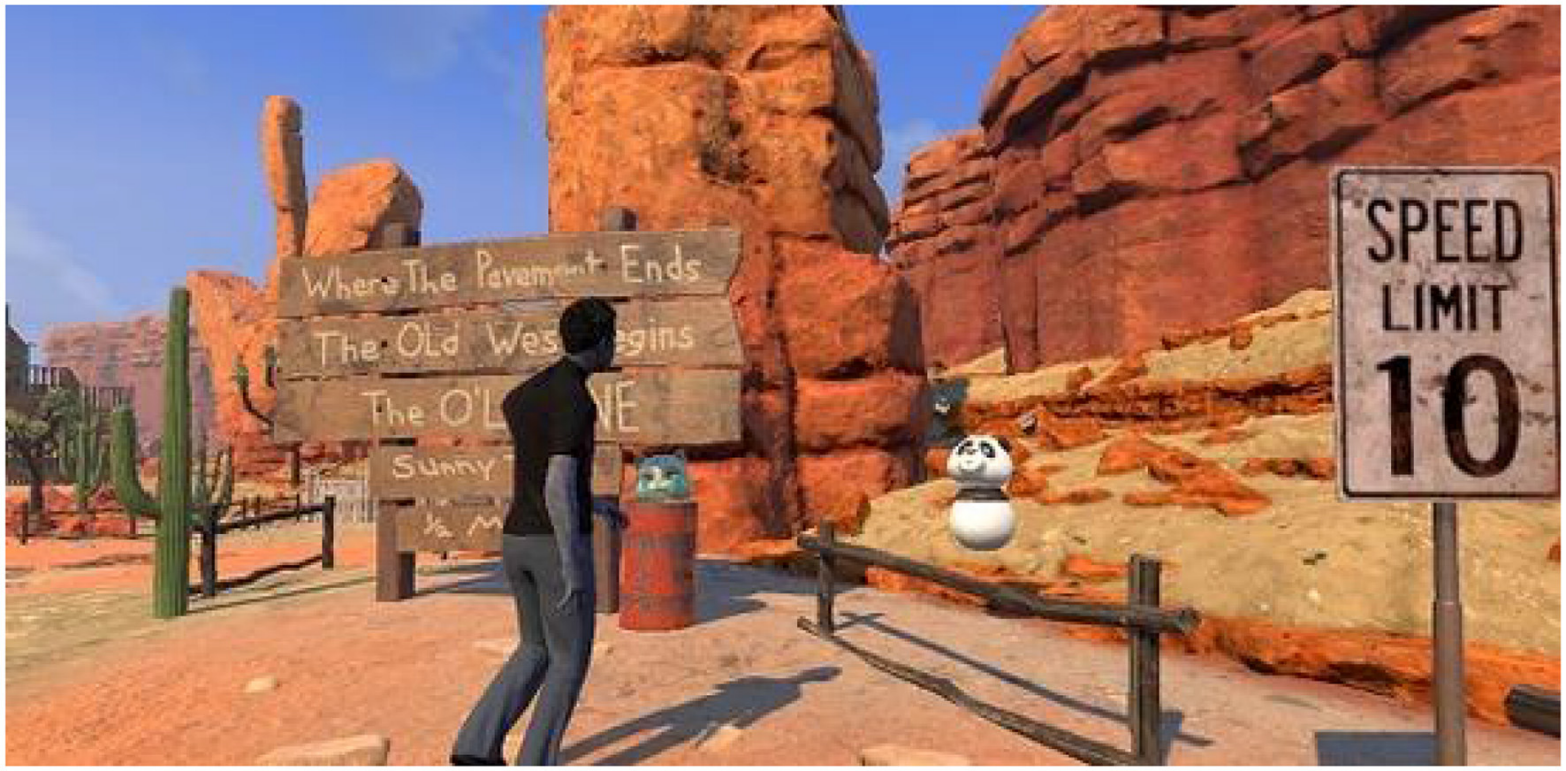
Screenshot of the VR game “Arizona sunshine.”

After finalizing the questionnaire, we collaborated with an offline VR experience store and invited customers who were willing to fill out the questionnaire to experience a VR game called “Arizona Sunshine,” which is one of the most popular VR games on Pico, a well-known Chinese VR game platform. The gaming experience live picture and scene of gaming live are shown in Figures 2, 3. We offered a subsidy of 20 RMB per person as an incentive. To collect more responses, we also invited undergraduate students who own a Head-Mounted Display (HMD) to experience the “Arizona Sunshine” VR game. We further expanded our participant base through online snowball sampling. This step was taken to explore the real-time experiences of playing VR games.

### 3.2 Subjects

Regarding the interview phase, we employed snowball sampling to recruit 25 undergraduate students-−17 males and eight females—from various universities across Guangdong Province, China. As for questionnaire, a total of 320 responses were collected. However, upon review, we identified and excluded 13 surveys due to participants being under the age of 18, which may have affected their comprehension of the questions and their ability to provide informed responses. Consequently, the dataset for statistical analysis was comprised of 307 valid responses. Table 3 provides a summary of the participants' demographic information.

**Table 3:** Demographic information of participants.

**Demographic categories**	**Range**	**Percentage**
Gender	Male	54.27%
	Female	45.73%
Year	18–30	55.05%
	31–43	42.02%
	44 or above	2.93%
Consumption per month	< 1,000	12.7%
	1,000–2,000	48.53%
	2,000–3,000	26.38%
	>3,000	12.38%
Prior experience of playing VR game	Never	52.44%
	Occasionally	45.73%
	Regularly	1.83%

### 3.3 Descriptive analysis

After collecting the data, descriptive analysis was conducted to understand the basic characteristics of the constructs (see Table 4). The average score of SOFS is the highest at 10.2541, which is higher than the evaluation score of SOFI. Moreover, SOFI has the widest score range, from 3 to 15, and the largest standard deviation of 3.31241, indicating that its score distribution is the most dispersed. In contrast, SOFS has the smallest standard deviation of 2.22301, showing that the scores are more concentrated. This suggests that participants rate the familiarity with the current VR game scenes higher than the familiarity with interactions. The average score of FE is the lowest at 7.9967. The average scores of SOE and PC are close, at 8.2671 and 8.3779, respectively, but PC has a larger standard deviation of 3.42529, implying greater variability in its scores. Although CINT has the highest average score at 8.8599, its standard deviation is also relatively large at 3.49929, indicating a higher degree of score dispersion.

**Table 4:** Descriptive analysis.

	***N***	**Minimum value**	**Maximum value**	**Mean**	**Standard deviation**
SOFS	307	4.00	14.00	10.2541	2.22301
SOFI	307	3.00	15.00	8.0912	3.31241
SOE	307	3.00	15.00	8.2671	2.94841
FE	307	3.00	15.00	7.9967	3.19364
PC	307	3.00	15.00	8.3779	3.42529
CINT	307	3.00	15.00	8.8599	3.49929

SOE, sense of embodiment; CINT, continued intention to play; FE, flow experience; SOFI, sense of familiarity with the interactivity; PC, perceived cost; SOFS, sense of familiarity with the scene.

### 3.4 Reliability and validity test

Then, this study meticulously assessed the reliability, convergent validities, and discriminant validities of the constructs. The findings indicate that all item loadings exceeded the anticipated threshold (>0.6), as detailed in Table 5, thereby confirming that the reliability standards were met. The Cronbach's alpha values were robust, with a minimum of 0.844 and all others surpassing the benchmark of 0.7, as advocated by Nunnelly (1978). This signifies that the reliability criteria were also fulfilled at the construct level.

**Table 5:** Reliability and validity of the scales.

**Variables**	**Items**	**Factor loading**	**Cronbach's α**	**AVE**	**CR**
CINT	CINT1	0.881	0.885	0.813	0.929
	CINT2	0.916			
	CINT3	0.908			
SOFS	SOFS1	0.851	0.844	0.762	0.906
	SOFS2	0.902			
	SOFS3	0.864			
FE	FE1	0.894	0.847	0.766	0.908
	FE2	0.862			
	FE3	0.869			
SOE	SOE1	0.892	0.871	0.795	0.921
	SOE2	0.898			
	SOE3	0.884			
SOFI	SOFI1	0.873	0.903	0.774	0.932
	SOFI2	0.882			
	SOF13	0.871			
	SOFI4	0.874			
PC	PC1	0.873	0.853	0.773	0.911
	PC2	0.891			
	PC3	0.872			

SOE, sense of embodiment; CINT, continued intention to play; FE, flow experience; SOFI, sense of familiarity with the interactivity; PC, perceived cost; SOFS, sense of familiarity with the scene.

Factor analysis further revealed that the factor loadings for the variables were above the recommended level of 0.8, aligning with the accepted criterion (>0.7). To evaluate convergent validity, we employed composite reliability (CR) and average variance extracted (AVE) metrics. The CR values for all constructs surpassed the threshold of 0.7, and the AVE values, which indicate the degree of variance captured by the constructs in relation to their indicators, were all above 0.5, in line with the guidelines set forth by O'Rourke and Hatcher (2013) and Hair et al. (2022). Table 6 illustrates that the average variance extracted for each construct exceeded 0.5, signifying that more than 50% of the variance in the indicators was attributed to their respective constructs.

**Table 6:** Convergent validity and discriminant validity tests.

	**SOE**	**CINT**	**FE**	**SOFI**	**SOFS**	**PC**
SOE	**0.891**					
CINT	0.716	**0.902**				
FE	0.728	0.705	**0.875**			
SOFI	0.786	0.825	0.785	**0.880**		
SOFS	0.756	0.763	0.738	0.863	**0.873**	
PC	0.723	0.75	0.724	0.794	0.757	**0.879**

SOE, sense of embodiment; CINT, continued intention to play; FE, flow experience; SOFI, sense of familiarity with the interactivity; PC, perceived cost; SOFS, sense of familiarity with the scene. The bold values are the square root of the AVE for each dimension.

In this study, as evidenced in Table 6, the square root of the AVE for each dimension was found to be greater than the correlation coefficients of the related variables. This finding underscores a high level of discriminant validity, indicating that the constructs are sufficiently distinct from one another. Collectively, these results demonstrate that the study's measures of reliability, as well as convergent and discriminant validities, are adequately addressed.

### 3.5 Structure model

**Figure 4:**
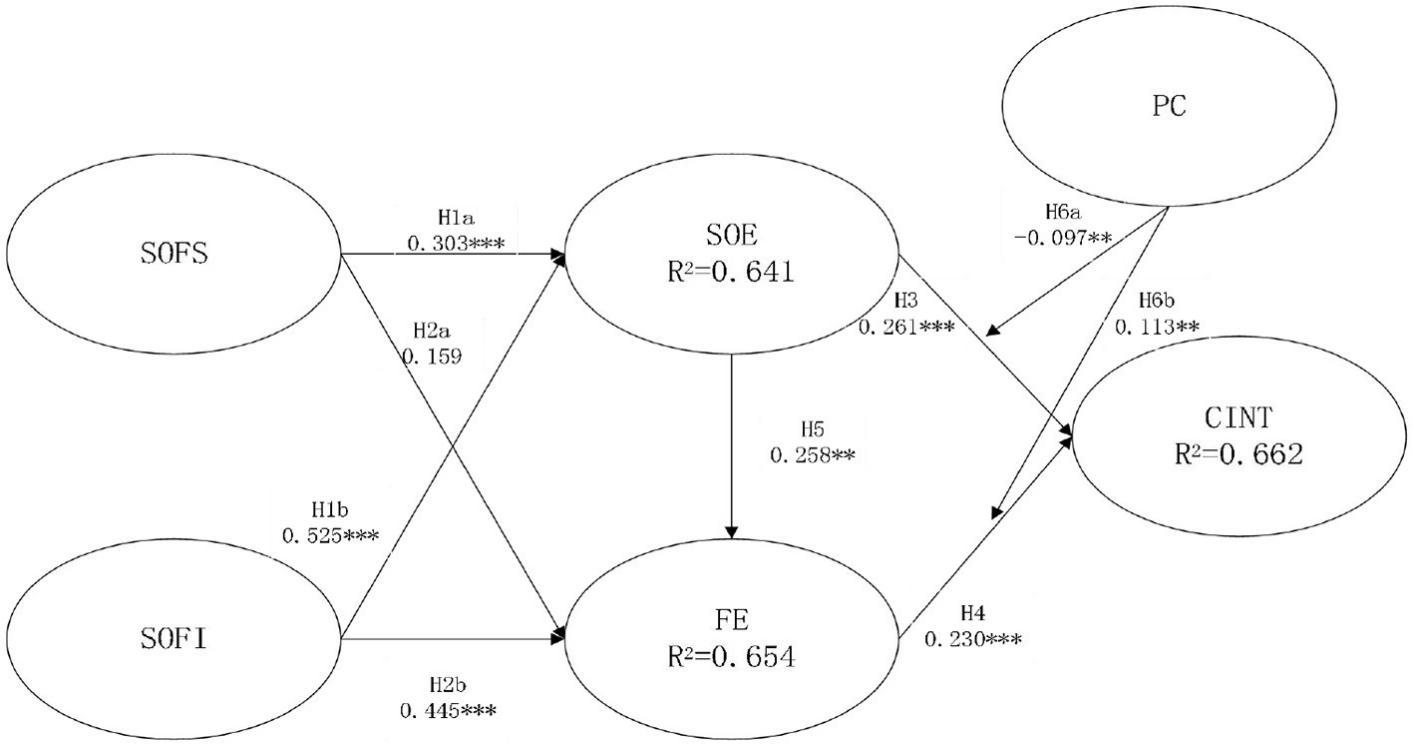
Structural model test results. *p < 0.05; **p < 0.01; ***p < 0.001 (two-tailed test). SOE, sense of embodiment; CINT, continued intention to play; FE, flow experience; SOFI, sense of familiarity with the interactivity; SOFS, sense of familiarity with the scene.

Partial Least Squares Structural Equation Modeling (PLS-SEM) was applied in this study to examine the relationships among the constructs because it is particularly suitable for handling small sample data that is not normally distributed (Gaomiao, 2021). Due to the limitations of time and economic conditions in this study, which prevent the acquisition of a large sample, the use of PLS-SEM not only enables the effective use of limited data to assess complex theoretical relationships but also reveals how different factors collectively influence the users' immersive experience and continued intention to play. Meanwhile, it contains the bootstrapping re-sampling technique, utilizing 5,000 sub-samples, was instrumental in determining the significance of the path coefficients between the constructs (Chin, 1998). The structural model, depicted in Figure 4, illustrates the interrelations among various constructs and elucidates these relationships through the R-square values. The explained variance (*R*^2^) for the sense of familiarity with the scene and interactivity was 64.8% and 65.5% for the SOE and flow experience, respectively; the *R*^2^ for the impact of SOE and flow experience on the continued intention to play was 58.4%.

Regarding path significance, bootstrapping serves as a robust non-parametric statistical technique that evaluates the accuracy of robust estimates for standard errors and confidence intervals of population parameters through re-sampling with replacement (Davcik, 2013). Table 7 presents the outcomes of the structural model analysis, encompassing path coefficients, standard errors, *t*-values, and *p*-values for the hypotheses. Hypotheses H1a and H1b propose that the sense of familiarity with the scene and interactivity positively influences the SOE. As evidenced in Figure 4, the SOF with the scene (β = 0.303, *p* < 0.001) and interactivity (β = 0.525, *p* < 0.001) exhibited significant positive relationships with the SOE, substantiating the validity of H1a and H1b. Hypotheses H2a and H2b suggest that the SOF with the scene and interactivity positively correlates with the flow experience. While the SOF with interactivity positively influenced the flow experience (β = 0.445, *p* < 0.001), the SOF with the scene did not yield a significant effect (*p* > 0.05). Consequently, H2a is not supported, whereas H2b is supported. The results indicate that SOE (H3) and flow experience (H4) are positively associated with the continued intention to play (β = 0.261, *p* < 0.001; β = 0.230, *p* < 0.001, respectively). Lastly, H5, which posits a significant positive relationship between SOE and flow experience, is confirmed with a β value of 0.258 and a *p*-value of < 0.06.

**Table 7:** Bootstrapping results.

	**Path coefficient**	**SE**	***t*-statistics**	***p*-value**	**Results**
SOE -> CINT (H3)	0.261	0.060	4.367	0.000	Supported
SOE -> FE (H5)	0.258	0.074	3.481	0.001	Supported
FE -> CINT (H4)	0.230	0.064	3.584	0.000	Supported
SOFI -> SOE (H1b)	0.525	0.075	7.035	0.000	Supported
SOFI -> FE (H2b)	0.445	0.088	5.039	0.000	Supported
SOFS -> SOE (H1a)	0.303	0.084	3.589	0.000	Supported
SOFS -> FE (H2a)	0.159	0.085	1.876	0.061	Not supported
SOE × PC-> CINT (H6a)	−0.097	0.033	2.909	0.004	Not supported
FE × PC -> CINT (H6b)	0.113	0.035	3.249	0.001	Supported

SOE, sense of embodiment; CINT, continued intention to play; FE, flow experience; SOFI, sense of familiarity with the interactivity; PC, perceived cost; SOFS, sense of familiarity with the scene.

To find out how perceived cost affects other factors, Smart PLS was used to calculate scores for a model that initially did not include the “perceived cost” variable. After that, the scores for the interaction terms were recalculated after adding “perceived cost,” allowing for a comparison between the two situations. According to the guidelines from Baron and Kenny (1986), a moderating effect of “perceived cost” is confirmed if the path coefficient (β) of the interaction term is statistically significant (*p* < 0.05). In this study, the path coefficient for the moderating effect was very significant (*p* < 0.01), as shown in Table 5.

To better understand the size of the moderating effect, the explained variance (*R*^2^) was compared between the two situations. When “perceived cost” was included, the *R*^2^ increased from 0.656 to 0.662. This increase indicates an improvement of about 0.06 in the explained variance, or more specifically, a 9.1% increase compared to the original model. Additionally, perceived cost had a negative effect on the relationship between SOE and the intention to keep playing (β = −0.097), while it positively influenced the effect of flow experience on the intention to continue engaging with VR games (β = 0.113). As a result, H6a is not supported, while H6b is supported.

### 3.6 Importance-performance matrix analysis

**Figure 5:**
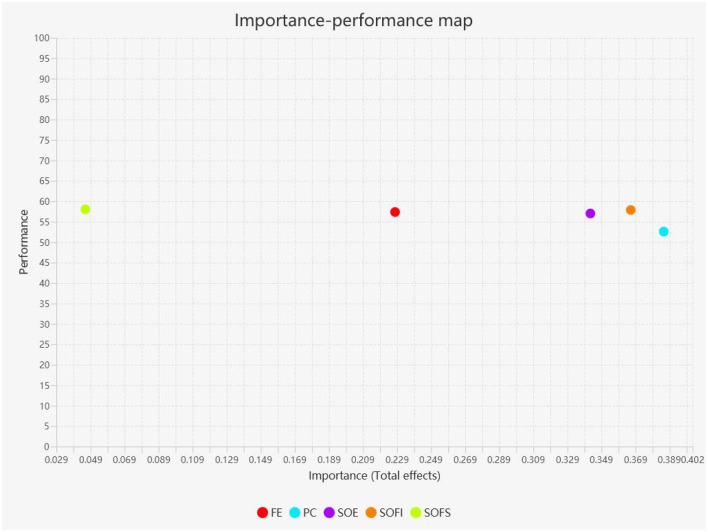
IMPA analysis. SOE, sense of embodiment; CINT, continued intention to play; FE, flow experience; SOFI, sense of familiarity with the interactivity; PC, perceived cost; SOFS, sense of familiarity with the scene.

This article uses the Importance-Performance Map Analysis (IPMA) to verify the importance and performance of constructs (Hair et al., 2022), with the results shown in Figure 5. The importance SOFI is 0.366, with a performance of 57.883; the importance of PC is 0.385, with a performance of 52.598; the importance of SOE is 0.342, with a performance of 57.029. This indicates that SOFI, PC, and SOE are significant in influencing users' CINT, and they also perform well in implementation. In particular, compared to SOFS, SOFI's performance is not as good as SOFS (58.049), but its importance is significantly higher than SOFS (0.046). This suggests that while SOFI may be slightly less effective in terms of performance in influencing users' continuous intention to consume, its importance is markedly higher than SOFS. This could mean that although SOFI may not be as directly effective as SOFS in practical applications, it plays an indispensable role in the process of forming users' continuous intention to consume. SOFI may provide a unique perspective or value that plays a key role in the user's decision-making process, even if its efficiency in actual execution or operation is not as good as SOFS. Furthermore, the difference between importance and performance may also point to a potential improvement point for SOFI in user experience.

## 4 Discussion

Understanding the key factors that influence users' continued intention to play VR games is crucial for VR game designers and marketers. Our study diverges from previous research that focused on the role of perceived ease of use or perceived quality in the acceptance of VR games (Kosa et al., 2023; Sagnier et al., 2020), by examining the impact of factors such as SOF, SOE, and flow experience on enhancing the continued intention to engage with VR games.

In this study, we found through empirical analysis that the SOE has a significant positive impact on their flow experience and continued intention to play VR games. This finding echoes the literature reviews by Leveau and Camus (2023) and Flavián et al. (2019), who emphasized the central role of SOE in VR gaming behavioral intentions. Additionally, our results align with the studies by Su et al. (2016) and Zhou (2019), indicating that the flow experience is a key factor driving users' continued intention to VR games. We speculate that VR technology, by enhancing the physical alignment between users and their avatars, evokes empathy and creates a pleasant and immersive gaming experience that encourages ongoing play.

Our research also fills a gap in the study of factors affecting SOE, discovering a significant correlation between SOF with scenes and interactivity and SOE. Although scholars have suggested the potential impact of familiar environments on triggering embodied memory (Sutton and Williamson, 2014), and familiar memories can enhance SOE (Lei and Mou, 2021), this is the first study to directly explore the relationship between SOF and SOE. Through interviews with participants, we found that objects and movement mechanics in the virtual environment are key triggers for SOE, with many users associating virtual actions with their real-world experiences. Notably, contrary to expectations, the SOF with interactivity has a greater impact on the flow experience than the SOF with the scene, indicating that interactivity, more than the scene, can stimulate users' immersion in VR games.

In terms of perceived cost, our study's results are consistent with the research by Hsu and Lin (2016) and Liu et al. (2015), showing that perceived cost has a negative impact on the relationship between SOE and continued gaming intention. Despite the decreasing cost of VR headset devices, the perceived value of VR experiences may not yet meet users' expectations, affecting their ongoing engagement with VR games. However, surprisingly, perceived cost positively moderates the relationship between the flow experience and continued gaming intention, contradicting the findings of Hsu and Lin (2016) but supporting the results of Wang et al. (2018) and Vishwakarma (2020). This positive effect may stem from the increased benefits of immersion, perceived control, and enjoyment outweighing the costs associated with continued VR gaming. The diversity in VR device prices on the market, along with the widespread availability of 5G signals, which are essential for VR gaming, may promote a better VR experience, making VR technology more accessible. Moreover, most users belong to Generation Z, who are more adept at adopting and adapting to new devices, reducing their difficulties with headset usage and enhancing their enjoyment of VR games. Therefore, for this generation, perceived cost positively moderates the impact of the flow experience on their continued intention to engage with VR games.

### 4.1 Theoretical implications

The theoretical implications of this study suggest a significant expansion of the Extended Technology Acceptance Model (TAM) framework by incorporating the concepts of Sense of Embodiment (SOE), Sense of Familiarity (SOF), and flow experience, which are crucial for understanding user behavior in VR gaming. The findings underscore the pivotal role of SOE in shaping users' behavioral intentions within the VR gaming context, aligning with and reinforcing previous literature. Additionally, the study redefines the impact of SOF by highlighting the importance of interactivity over the familiarity of the scene itself, which could lead to a reevaluation of virtual environment design. It also supports the applicability of flow theory in VR gaming, emphasizing the immersive nature of VR as a key driver of continued engagement. Furthermore, the nuanced view of perceived cost as both a barrier and an enhancer of the gaming experience, especially for tech-savvy generations like Generation Z, adds depth to our understanding of cost's impact on VR gaming intentions.

### 4.2 Practical implications

Practically, these findings have several implications. Gaming companies must understand user behavior in VR to innovate effectively. The critical role of SOE in user experiences suggests that enhancing this aspect can give companies a competitive edge. Engaging with users through feedback can lead to creative solutions that satisfy and delight, which is essential for a game's success. Companies should also focus on creating environments that are familiar and interactive to foster a sense of embodiment and flow. Especially for SOF with interactivity, although is very important in user decision-making, if its performance can be improved, it may further enhance users' intention to play VR game. Therefore, gaming companies can focus on optimizing the implementation strategies of SOF with interactivity to improve its efficiency and effectiveness in practical applications. This includes designing games with recognizable elements and interactions that align with real-world logic. To attract users from traditional gaming platforms, VR companies should aim to reduce perceived costs. They can do this by collaborating with established franchises to leverage familiarity, simplifying the process of using VR headsets, and continuously lowering the costs of both hardware and software. These strategies can help broaden the appeal of VR games and encourage more people to transition from PC or mobile gaming to VR.

### 4.3 Limitation and future direction

While the research methodology of this study is robust and the topic compelling, there are several limitations to acknowledge. Firstly, due to the relatively small number of VR game users, the selection of volunteers in this study was not random, which may have introduced sampling bias. For instance, the majority of the participants were aged between 18 and 30 and were concentrated in specific regions, limiting the representativeness of the findings to other demographics or areas. This could potentially reduce the generalizability of the results. To enhance the precision and broad applicability of future studies, researchers should employ random sampling techniques to include participants from a diverse array of countries or regions. Additionally, further investigation into the impact of personal characteristics such as gender, age, monthly expenditure, and prior gaming experience would be beneficial.

Moreover, the current model does not compare the relative effectiveness of various scenes and interactivities in evoking a SOF. Therefore, subsequent experiments should be designed to ascertain which elements are most potent in triggering familiarity. By addressing these limitations, future research can offer a more comprehensive understanding of the factors influencing user engagement and intention to continue playing VR games.

## 5 Conclusion

This paper set out to scrutinize the impact of the SOF (SOF) in VR gaming, aiming to decipher the embodied responses and flow experiences of users within the VR environment. The model presented in this paper posits that SOF, encompassing both the game's setting and interactivity, exerts influence on users' SOE and flow experience, thereby subsequently shaping their intention to persist with VR gaming. The proposed model, bolstered by empirical evidence, offers profound insights into the factors driving users' sustained engagement with VR games. Specifically, this study delivers three significant contributions toward enhancing our understanding of the motivations behind continued VR gaming.

Firstly, by integrating embodied cognition into the discourse on users' intentions to continue playing VR games—a realm scarcely explored in existing literature—this study pioneers a novel avenue for research into VR gaming behavior. This innovative approach opens up new horizons for academic inquiry and practical application within the field of VR gaming.

Secondly, the study's findings are grounded in established theories and observable phenomena, elucidating the mechanisms through which a SOF with the game environment and interactivity can significantly influence the intention to continue playing, mediated by the SOE. Notably, the research reveals a particularly intriguing dynamic: the SOF with interactivity has a more pronounced positive impact on the flow experience than the SOF with the game's setting.

Thirdly, the study uncovers a nuanced role of perceived cost, demonstrating that it significantly and positively moderates the relationship between flow experience and the intention to continue gaming, while simultaneously exerting a negative moderation on the link between SOE and continued gaming intentions within the VR context. This distinctive finding addresses a notable gap in the literature regarding the moderating influence of perceived cost on the effects of SOE and flow experience on gaming intentions, thereby enriching our comprehension of user behavior in the realm of VR gaming.

## Data Availability

The raw data supporting the conclusions of this article will be made available by the authors, without undue reservation.
